# “The structure of the Type III secretion system export gate with CdsO, an ATPase lever arm”

**DOI:** 10.1371/journal.ppat.1008923

**Published:** 2020-10-13

**Authors:** Jaime L. Jensen, Shavait Yamini, Arne Rietsch, Benjamin W. Spiller

**Affiliations:** 1 Department of Pathology, Microbiology and Immunology, Vanderbilt University Medical Center, Nashville, TN, United States of America; 2 Department of Pharmacology, Vanderbilt University, Nashville, TN, United States of America; 3 Department of Molecular Biology and Microbiology, Case Western Reserve University, Cleveland, OH, United States of America; Purdue University, UNITED STATES

## Abstract

Type III protein secretion systems (T3SS) deliver effector proteins from the Gram-negative bacterial cytoplasm into a eukaryotic host cell through a syringe-like, multi-protein nanomachine. Cytosolic components of T3SS include a portion of the export apparatus, which traverses the inner membrane and features the opening of the secretion channel, and the sorting complex for substrate recognition and for providing the energetics required for protein secretion. Two components critical for efficient effector export are the export gate protein and the ATPase, which are proposed to be linked by the central stalk protein of the ATPase. We present the structure of the soluble export gate homo-nonamer, CdsV, in complex with the central stalk protein, CdsO, of its cognate ATPase, both derived from *Chlamydia pneumoniae*. This structure defines the interface between these essential T3S proteins and reveals that CdsO engages the periphery of the export gate that may allow the ATPase to catalyze an opening between export gate subunits to allow cargo to enter the export apparatus. We also demonstrate through structure-based mutagenesis of the homologous export gate in *Pseudomonas aeruginosa* that mutation of this interface disrupts effector secretion. These results provide novel insights into the molecular mechanisms governing active substrate recognition and translocation through a T3SS.

## Author summary

Many pathogenic Gram-negative bacteria utilize T3SS to export virulence factors in a well-regulated manner. Most component proteins of the T3SS are highly structurally conserved, capable of recognizing and secreting diverse effectors, which are recruited to the cytoplasmic sorting complex by chaperones. This work describes the molecular architecture of two essential components of a T3SS, identifies the interface between the components, and establishes the necessity of this interaction for effector secretion.

## Introduction

Bacterial pathogens secrete toxins and other effectors to promote virulence by subverting host processes and defenses through the evolution of specialized secretion systems (type I to type IX). The type III secretion system (T3SS) is among the most complex and is an essential virulence factor for many pathogenic Gram-negative bacteria, including *Bordetella*, *Chlamydia*, EHEC/EPEC, *Pseudomonas*, *Salmonella*, *Shigella*, and *Yersinia*. Although T3S effectors are generally not conserved across species, the secretion apparatus itself (the T3S injectisome), is well-conserved. In addition, the injectisome shares structural similarities with the flagellar T3SS, which bacteria utilize for motility [1].

Structural studies of the T3S injectisome and flagellum have significantly improved our understanding of T3SS structure and mechanism, and revealed this conserved nanomachine to be composed of several complexes [2]. Two concentric protein rings, the inner and the outer membrane ring complexes, span the bacterial membranes and form the basal body to which the needle docks [3]. The needle complex terminates in a translocon pore, which forms the final conduit into the target cell [4–6]. Two additional complexes, an inner membrane anchored export apparatus and cytoplasmic sorting complex, are less well-characterized [7–12].

**Fig 1:**
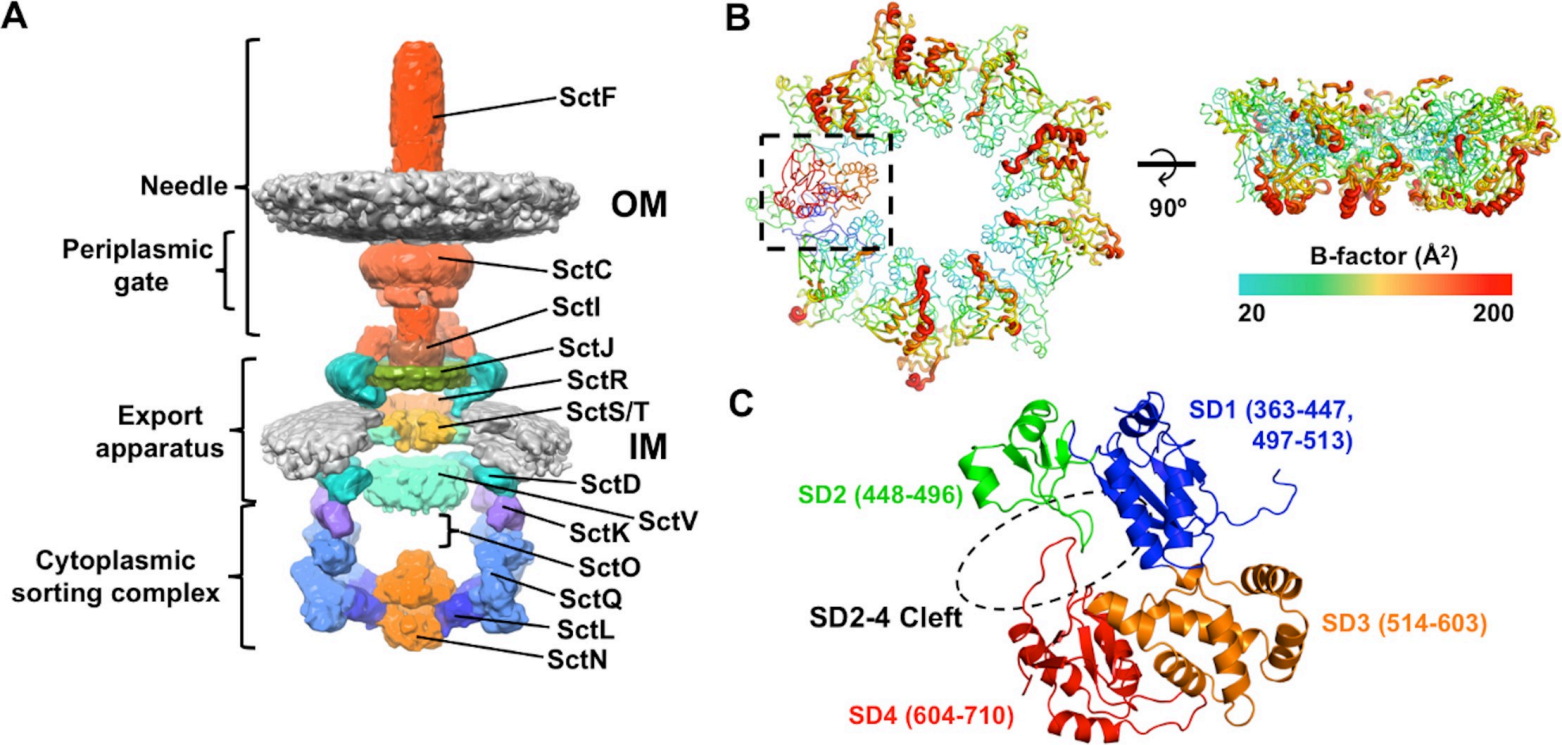
Overview of the T3S injectisome and the homo-nonameric ring of CdsV_C_. (A) Cut-through view of the *Salmonella* injectisome, from *in situ* tomography (EMDB 8544; [10]). Individual or oligomerized components are colored and labeled according to the secretion and cellular translocation (Sct) unified nomenclature [47]. The locations of the outer membrane (OM), needle filament, periplasmic gate, export apparatus, inner membrane (IM), and cytoplasmic sorting complex are indicated. The major component of the export apparatus (SctV or CdsV, from *Chlamydia*, in cyan) and the central stalk protein (SctO or CdsO), characterized in this work, are indicated. SctO is not visible in the 3D map of the injectisome. (B) Cartoon representation of bottom and lateral views of the CdsV_C_ structure, colored according to B-factor values. The lateral view is obtained by 90° rotation of the bottom view about the x-axis. One protomer of the CdsV_C_ ring is boxed and colored by subdomain, as shown in (C). (C) Subdomains of CdsV: subdomain 1, blue; subdomain 2, green; subdomain 3, orange; and subdomain 4, red. Residues corresponding to subdomain boundaries are indicated.

A recent, 17 Å *in situ* structure, obtained by cryo-electron tomography, showed the entirety of the T3SS from *Salmonella enterica* serovar Typhimurium and revealed the overall architecture of the cytosolic components of an intact T3SS (Fig 1A) [10,13]. The export apparatus is composed of a central, nonameric, ring-shaped inner membrane protein, termed the export gate (CdsV in *Chlamydia* species and SctV in universal nomenclature [14]). The export gate engages additional inner membrane proteins SctR, SctS, SctT, and SctU to form the export apparatus [10]. The sorting platform (composed of SctL, SctQ, and SctK) forms a cage around the export apparatus and links the inner membrane protein complex to the ATPase (SctN) [4,15,16]. The export gate is known to undergo an opening and closing of the cleft between subdomains 2 and 4 (the SD2-4 cleft) and closing of this cleft promotes substrate release [17–19]. The export gate does not directly engage the sorting complex, but is linked to the ATPase by SctO [8], an ~140 Å coiled-coil that is structurally similar to the central stalk proteins of the rotary ATPases- the F_1_-ATPase γ-subunit and the V_1_-ATPase D subunit [7,20,21]. SctO is essential for substrate secretion [22–24].

The T3S ATPase itself is structurally related to the F- and V-type ATPases and has been proposed to function with a similar rotary catalytic mechanism wherein a coiled-coil subunit (SctO) engages the asymmetric pore of the homo-hexameric ATPase and SctO rotates during ATP hydrolysis cycles, shifting interactions to neighboring ATPase subunits coincident with ATP hydrolysis [1,25]. In T3SS, SctO is the key link between the export gate and the ATPase and is poised to transmit mechanical force between the ATPase and the export gate. A recent cryo-EM structure of the T3SS ATPase:central stalk complex from *E*. *coli* (EscN:EscO) revealed a single EscO extending away from EscN at an ~70° angle, and comparison of the EscN homo-hexamer and the EscO-bound structures suggests a rotary catalytic mechanism similar to that observed for the F- and V-type ATPases, in which EscO rotates during ATPase catalysis [25].

Presented in this manuscript are structures of the C-terminal region of the export gate from *Chlamydia pneumoniae* (CdsV), both in an unliganded form and when bound to residues 25–110 of the *Chlamydial* SctO (CdsO). These structures show that CdsO engages CdsV in a cleft between adjacent subunits and influences the configuration of the SD2-4 cleft, thus revealing how the ATPase may control substrate release by rotating CdsO.

## Results

### Structure of CdsVc

We determined the crystal structure of the CdsVc (CdsV C-terminal region) homo-nonameric ring assembly and refined the structure to 2.8 Å (Table 1; Fig 1B and 1C; PDB 6WA6). The crystallized protein contains residues 345–710 of CdsV from *Chlamydia pneumoniae*; several N- and C-terminal residues from most monomers could not be resolved from electron density difference maps. S1 Table and Table 1 describe the statistics of the refined models and the contents, or completeness, of those models. The amino terminal region, approximately residues 1–345 of CdsV and other export gate homologs, are predicted to contain 6 transmembrane helices, which anchor CdsV to the inner membrane. CdsV_C_ displays the same fold as homologs MxiA from *Shigella flexneri*, InvA from *Salmonella typhimurium*, and FlhA monomers from *S*. *typhimurium*, *Bacillus subtilis*, and *Helicobacter pylori*, with four distinct subdomains (subdomains 1–4) (Fig 1C) [17,26–30]. Monomers of CdsV_C_ align with RMS deviations of 0.26–1.68 Å; the primary differences across the nine subunits exist in subdomains 2 and 4, and in particular, the cleft formed between subdomain 4 of neighboring CdsV protomers, as evidenced by the high B-factors observed in the structure (Fig 1B, S6A Fig). The closed, planar ring is stabilized by the buried surface area between subdomains 1 and 3, with an average total interaction area of 1127 Å^2^, as well as several salt bridges and hydrogen bonds between conserved residues (S2 and S3 Figs), as noted for MxiA [26]. The CdsV_C_ nonamer has an inner pore diameter of ~60 Å, with the total diameter of the ring ~170 Å.

**Table 1:** Data collection and refinement statistics.

	CdsV_C_	CdsV_C_:CdsO
Data collection		
Beamline	APS 24-ID-C	APS 21-ID-D
Space group	*P2*_*1*_*2*_*1*_*2*_*1*_	*P2*_*1*_*2*_*1*_*2*_*1*_
Cell dimensions		
*a*, *b*, *c* (Å)	66.65, 280.44, 290.41	156.41, 206.61, 280.59
**α**, **β**, **γ** (°)	90, 90, 90	90, 90, 90
Wavelength	0.97910	0.97850
Resolution (Å)	126.27–2.80 (2.85–2.80)	52.27–4.62 (4.70–4.62)
R_merge_	0.089 (1.109)	0.141 (1.726)
*I/****σ****I*	15.6 (1.9)	8.1 (1.4)
CC1/2	0.999 (0.718)	0.998 (0.570)
Completeness (%)	99.4 (99.2)	99.3 (99.9)
Redundancy	6.0 (6.0)	7.5 (8.0)
Refinement		
Resolution (Å)	100.9–2.8 (2.9–2.8)	52.2–4.62 (4.8–4.62)
No. reflections	134496 (13227)	50242 (4970)
*R*_*work*_*/R*_*free*_ (%)	19.6/24.4	24.1/28.6
No. atoms	24992	29937
Protein	24856	29937
Ligands	78	
Water	58	
Mean *B*-factors (Å^2^)	95.94	307.34
Protein	96.02	
Ligands	104.18	
Water	53.02	
R.m.s. deviations		
Bond lengths (Å)	0.009	0.004
Bond angles (°)	1.13	0.69
Ramachandran		
Outliers	0.00	0.00
Allowed	4.10	3.73
Favored	95.90	96.27
PDB ID	6WA6	6WA9

Values in parentheses represent the highest resolution shell.

Residues lining the inner surface of the ring, which correspond to subdomain 3, are highly evolutionarily conserved across prokaryotes with T3S injectisome machinery (S2 and S4 Figs) and flagellar T3S, while residues along the outside surface (subdomain 2) are highly variable. This suggests a conserved functional role, such as substrate secretion, for residues lining the pore, while divergence of the outer surface may allow the export gate platform to form multiple species-specific interactions. Indeed, deletion of subdomain 2 of MxiA did not abolish effector secretion, but did impact secretion of translocon components [26]. As was shown for MxiA, conserved residues lining the CdsV_C_ pore also include several lysines and arginines (S4 and S5 Figs), which are critical for secretion [26].

### Structure of the CdsV_C_:CdsO complex and oligomer assembly

**Fig 2:**
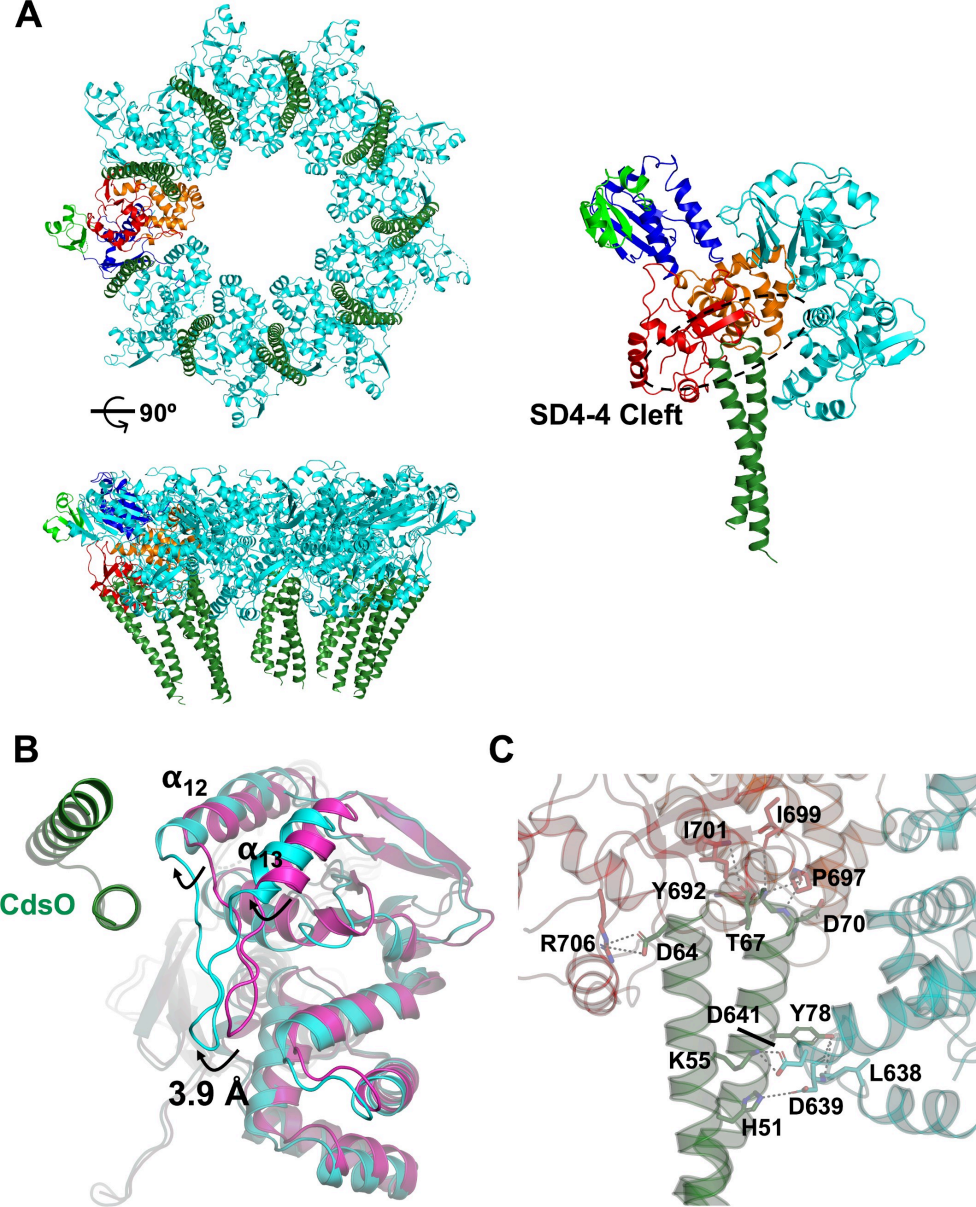
The CdsV_C_:CdsO assembly. (A) Bottom and lateral views of the 9:9 assembly of CdsV_C_ (cyan) with CdsO (green), with an enlarged view of a 2:1 CdsV_C_:CdsO complex showing the CdsO binding site between adjacent CdsV_C_ protomers (the SD4-4 cleft). One CdsV_C_ protomer is colored by subdomain as in Fig 1C. The lateral view is obtained by 90° rotation of the bottom view about the x-axis. In the rightmost panel, β-strand 11 is below the top portion of the ellipse. (B) Bottom view of the superposition of unbound CdsV_C_ (magenta) with CdsO-bound CdsV_C_ (cyan). The movement of α-helices 12 and 13 and the loop connecting them, between the unbound and bound states, is highlighted. (C) Residues at the CdsV_C_:CdsO interface are labeled and displayed as sticks, with dashed lines indicating salt bridges or H-bonds.

We also determined the crystal structure of CdsV_C_ in complex with a portion of CdsO and refined the structure to 4.6 Å (Table 1; Fig 2; PDB 6WA9). The CdsO protein was truncated to residues 25–110 to facilitate crystallization of the complex. Although determined at a relatively low resolution, high-resolution structures of CdsV_C_ and the 84% identical CdsO from *Chlamydia trachomatis*, for which a high resolution structure exists [31], greatly simplified structure determination and interpretation. This structure defines the structural organization of the export gate bound to SctO. Several residues on both termini of CdsO_25-110_ could not be modeled, due to the limited resolution and poorly resolved electron density in the area (S1 Table). Most notably, we observed CdsO_25-110_ positioned within a large cleft at the interface between two CdsV_C_ protomers, specifically, between subdomain 4 of adjacent subunits (the SD4-4 cleft, see Fig 2A). In our crystals, a 1:1 stoichiometry between CdsV_C_ and CdsO_25-110_ is observed, and saturation of CdsV_C_ in this way likely aided crystallization. CdsO_25-110_ binding is mediated largely through electrostatic interactions and stabilized by the buried surface area of each face of the CdsO_25-110_ coiled-coil with one side of the CdsV_C_ monomer (Fig 2B and 2C). The interacting residues on CdsV are fairly well conserved (S2 and S4 Figs and Fig 2C), suggesting that a similar interaction may occur in other homologs. The average total interaction area of one CdsO_25-110_ with a CdsV_C_ dimer is ~900 Å^2^.

Large-scale architectural rearrangements were not observed in CdsV_C_ upon binding CdsO_25-110_ (Fig 2B, S6A Fig); instead, small conformational differences were identified primarily within subdomain 4. Helices 12 and 13 of CdsV_C_, connected by an extended loop, were displaced by an average of 3.9 Å and 9.5° when bound to CdsO_25-110_, which draws subdomain 4 further into the SD4-4 cleft to stabilize CdsO_25-110_. Comparison of CdsV_C_ with CdsV_C_:CdsO_25-110_ reveals that while both are similar to the “open” FlhA structures (S7 Fig), binding of CdsO_25-110_ in the SD4-4 cleft influences the SD2-4 cleft. The SD2-4 cleft is smaller, with helix 3 moving, on average, 5 Å toward subdomain 4 (S6B Fig), and the sheet formed by β-strands 8 and 11, adjusting by an average of 2.3 and 2.5 Å, respectively (S6C Fig). β-strand 11 binds the loop connecting each helix of the CdsO_25-110_ coiled-coil (Fig 2C); interestingly, this interface is mediated by H-bonds between backbone atoms of CdsV_C_ and CdsO_25-110_.

**Fig 3:**
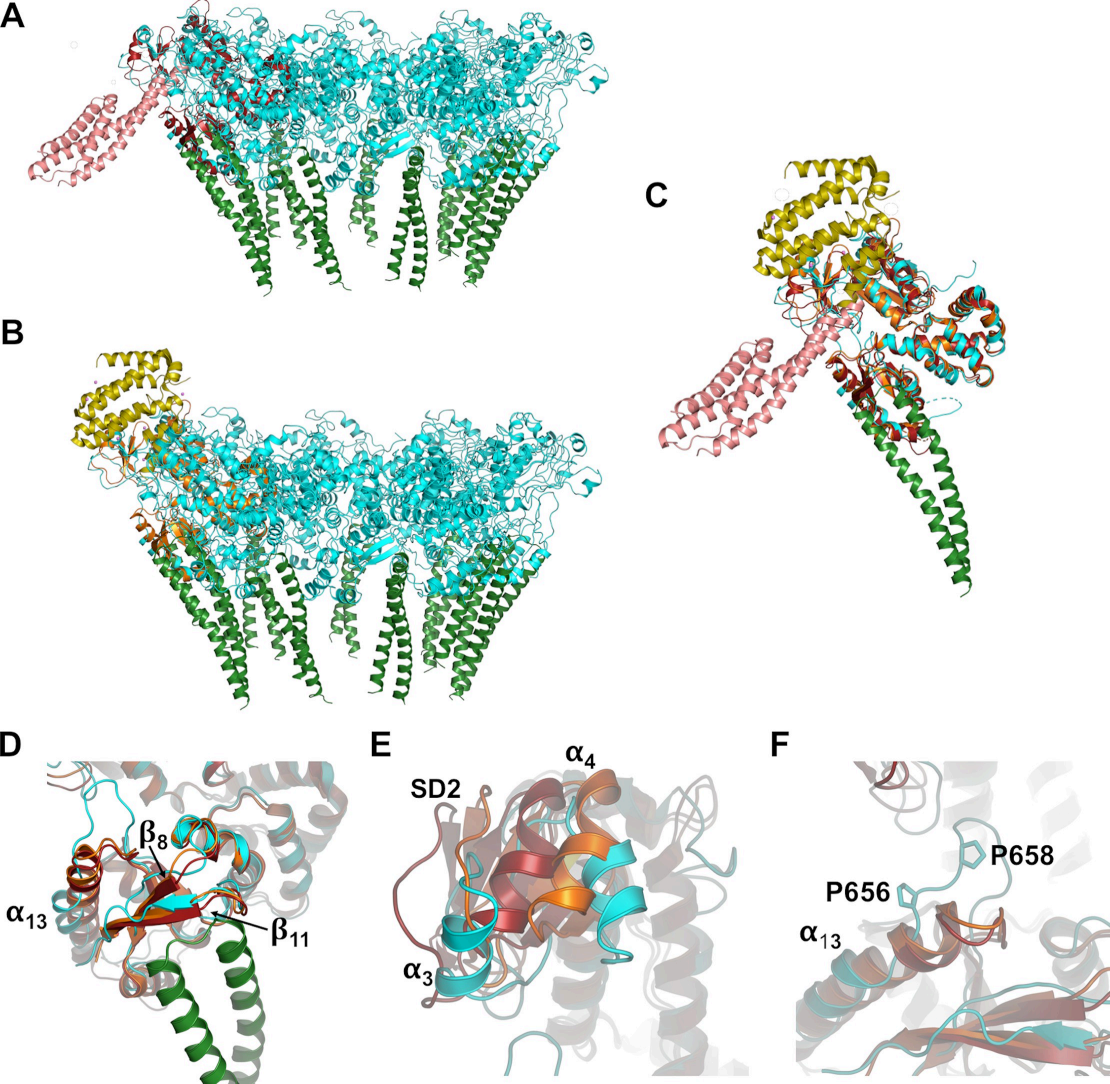
Superposition of export gate apparatus complexes. The CdsV_C_:CdsO ternary complex (cyan and green, respectively) is superimposed with structures of (A) flagellar CdsV ortholog FlhA (orange) with chaperone FliS (yellow; PDB ID 6CH3) and (B) FlhA (red) with FliT (salmon; 6CH2). (C) Superposition of FlhA:FliS and FlhA:FliT with CdsV_C_:CdsO. The FlhA monomers align to CdsV_C_ with RMS deviations of 1.76 and 1.59 Å, respectively. (D) β-strands 8 and 11 are only slightly shifted, in comparison of the CdsV_C_:CdsO (cyan) and chaperone-bound FlhA structures (red and orange). (E) α-helix 4 of chaperone-bound FlhAs adjust considerably to accommodate cargo, and α-helix 3 is disordered in these structures. (F) In *Chlamydia*, α-helix 13 is disrupted by P656 and P658; this helix is extended by ~2 turns in the chaperone-bound FlhA structures.

Two chaperones, FliT and FliS, have been shown to associate with the flagellar CdsV homolog FlhA [18]; these chaperones bind in the SD2-4 cleft to the “open” state of FlhA (S7 Fig) (17) slightly displacing β-strands 8 and 11 (Fig 3D). The binding of CdsO_25-110_ results in a structure more similar to the chaperone-bound structures than to a “closed” MxiA or FlhA structure, as reported in Inoue *et al*, 2019 (S7B–S7F Fig) (no such structure has been observed for CdsV) [32]. These binding clefts thus appear functionally linked, wherein binding at one site may promote binding at the other site. Helices 12 and 13 of subdomain 4 shift considerably into the region that forms the SD2-4 cleft when in the “closed” form (S7E and S7F Fig), which would occlude or restrict binding of CdsO. These regions are somewhat flexible in the absence of ligands, are the sites of the greatest structural differences between protomers, and the locations of missing electron density or high B-factors in both CdsV and MxiA [26]. Density for the loop connecting helices 12 and 13 is observed in the CdsV_C_:CdsO_25-110_ complex but is unresolved or only partially resolved in the structure of CdsV_C_ alone, despite this structure being at higher resolution than the CdsV_C_:CdsO_25-110_ complex, further supporting the idea that these clefts are flexible and become more stable when ligands are bound.

In FlhA, residues 621–641 form a helix (part of subdomain 4) that shifts position to allow for substrate binding (Fig 3F) [18,28]. In *Chlamydia*, this helix is shorter, as it is interrupted by Pro 656 and Pro 658, although these helix-breaking residues are not conserved in T3SS from other organisms (Fig 3F and S4 Fig). These structural changes would significantly alter the chaperone-binding site identified in FlhA such that *Chlamydia* may use a somewhat different substrate recognition strategy, likely still involving the large pocket that remains accessible in CdsV.

CdsO shares the coiled-coil motif with homologs from other injectisome and flagellar systems, and an existing structure for CdsO from *Chlamydia trachomatis* (PDB 3K29; [31]) was used for molecular replacement. The complex revealed the binding site of CdsO_25-110_, although, due to the limited resolution of the structure, only residues ~39–85 could be modeled per coiled-coil (S1 Table). The CdsO_25-110_ structure exhibits a key structural constraint. The loop connecting the two helices of the coiled-coil has a small, hydrophobic or uncharged residue midway between the helices, with its sidechain pointed parallel with the long axis of the coiled-coil (Thr 67 in *C*. *pneumoniae* CdsO and *C*. *trachomatis* CdsO; Val 64 in YscO from *Vibrio parahaemolyticus*; Gly 58 in FliJ). The presence of a small residue with its sidechain pointed along the coiled-coil results in a backbone- mediated interaction between CdsO_25-110_ and CdsV (Fig 2C). This interaction is formed between the last beta strand of CdsVc and the loop of CdsO_25-110_. Similar to the other YscO-like proteins, the two helices exhibit amphipathic packing of the sidechains central to the monomeric coiled-coil. Despite the common helix-loop-helix motif, YscO-like proteins display significant divergence in primary sequence (S8A and S8B Fig) and in protein size, as YscO-like proteins vary in length by as many as 40 residues. However, a commonality of the injectisome T3S SctOs is the conserved structure that is able to dock within the appropriate SctV and interact with the conserved sites at the base and sides of the SD4-4 cleft (Fig 2C). This interaction immobilizes both helices and the short loop between them.

To further evaluate the CdsV_C_: CdsO_25-110_ complex in the context of the full injectisome, the CdsO_25-110_ structure was manually extended to contain residues 1–162 of the 168 residues of full-length CdsO from *C*. *pneumoniae*, using the *C*. *trachomatis* CdsO as a template (S9A Fig). In this model, one helix of the coiled-coil extends beyond the other, as shown in the YscO, CdsO, and FliJ structures (S9B Fig). This positions a large positively-charged zone, flanked by a smaller, negatively-charged region, for interaction with the electrostatic ATPase pore.

### Mutations that disrupt the CdsV:CdsO interaction decrease secretion when introduced into PcrD in *Pseudomonas*

**Fig 4:**
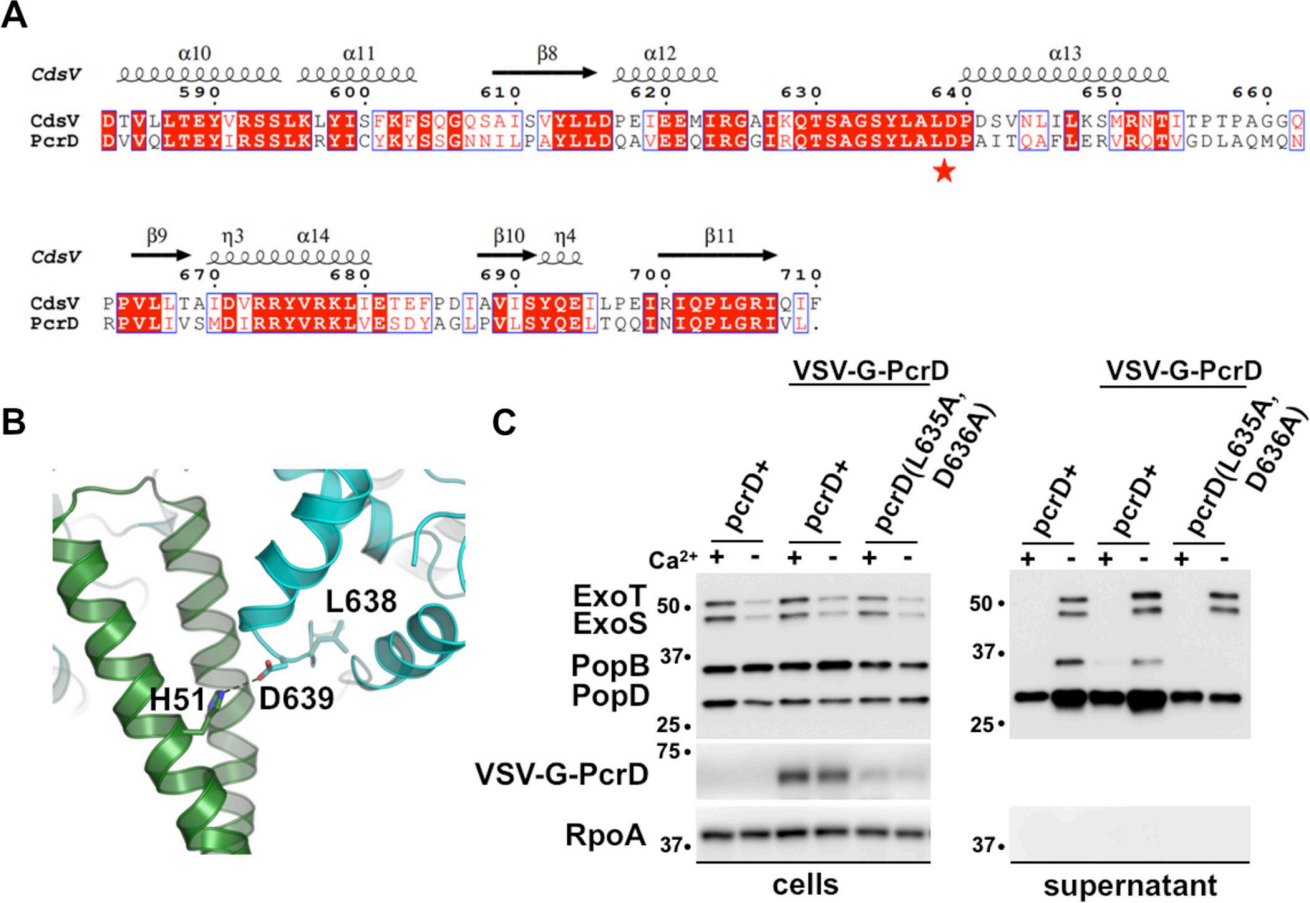
Disruption of effector secretion. (A) Sequence alignment of CdsV with the *Pseudomonas* ortholog PcrD. Residues identified for mutation are indicated with a red star. (B) Location of mutated residues within the CdsV_C_:CdsO complex. CdsV_C_ residues chosen for mutation are depicted as sticks. (C) T3S was assayed in *Pseudomonas* in the presence or absence of calcium (+EGTA, triggers effector secretion *in vitro*). Effector (ExoS and ExoT) and translocator (PopB and PopD) secretion of *Pseudomonas* expressing WT PcrD or the PcrD L635A/D636A double mutant were compared. Depressed levels of secreted translocators and effectors were observed for the double mutant. An epitope-tagged (VSV-G) version of PcrD was used to monitor protein levels in the cell pellet fraction. RNA polymerase alpha (RpoA) served as a fractionation control.

To functionally assess both the importance of the CdsV:CdsO interaction and its conservation in other T3SS, two structure-guided CdsV mutations were designed to disrupt the CdsV:CdsO interface. Mutations of L638 and D639 of CdsV_c_ to alanine abrogate binding between CdsV_c_ and CdsO_25-110_, as measured by isothermal titration calorimetry (S10A and S10B Fig). The CdsV_C_: CdsO_25-110_ complex has a Kd of 28 ± 3 μM, whereas the L638A/D639A mutant does not appear to bind CdsO (S10A and S10B Fig). D639 forms a salt bridge with H51 of CdsO_25-110_ (Figs 2C and 4B), which may account for the importance of this interaction. These mutations have a minimal effect on stability as WT CdsVc and L638A/D639A have melting temperatures of 58°C and 55°C, respectively (S10C Fig). These residues are located within a broadly conserved region in SctV proteins and are invariant between *C*. *pneumoniae* and *Pseudomonas aeruginosa* (Fig 4A and S4 Fig). The homologous mutations, L635A/D636A, were made in *Pseudomonas aeruginosa* (*pcrD*) and bacteria were evaluated for secretion competency. Presence of the effector proteins ExoT and ExoS, and translocator proteins PopB and PopD, in *Pseudomonas aeruginosa* PA01 *ΔexsE* culture medium was compared with the presence of secreted proteins in the L635A/D636A double mutant and in wild type PcrD (Fig 4C). As expected, ExoS and ExoT were detected in supernatant from WT PA01 Δ*exsE* containing the Ca^2+^ chelator EGTA, while PopB and PopD were detected in both the presence and absence of Ca^2+^ (Fig 4C) [33]. The L635A/D636A double mutant was partially defective for secretion. We note that mutant PcrD was also expressed at a lower level than an unmutated epitope tagged control (Fig 4C), such that while we cautiously suggest that the contribution residues 635 and 636 make to the PcrD-PcsO interaction is important for maintaining secretion, the reduced secretion could be due to an unrelated stability affect in PcrD that is not seen in CdsV.

## Discussion

Our structure of the CdsV_C_: CdsO_25-110_ complex provides, for the first time, molecular details of the interaction of an export gate apparatus with the central stalk protein of a T3SS. Two new findings stem from this structure. First, the structure reveals that CdsO binds in an inter-subunit cleft between subdomain 4 of adjacent protomers, rather than in the central pore of CdsV (Fig 2). This region borders the recently described binding site, between subdomains 2 and 4 of a single protomer, for chaperone-cargo complexes (Fig 3 and [18]). Second, we observed full occupancy of the CdsV_C_ binding sites by CdsO. The 9:9 stoichiometry of the CdsV_C_:CdsO interaction observed in our structure indicate that symmetric binding is possible; however, as others have shown, only one CdsO may bind the ATPase at a time. Finally, the CdsV_C_:CdsO_25-110_ complex allows a structural interpretation of mutations in SctV and SctO proteins that have been shown to alter secretion kinetics.

**Fig 5:**
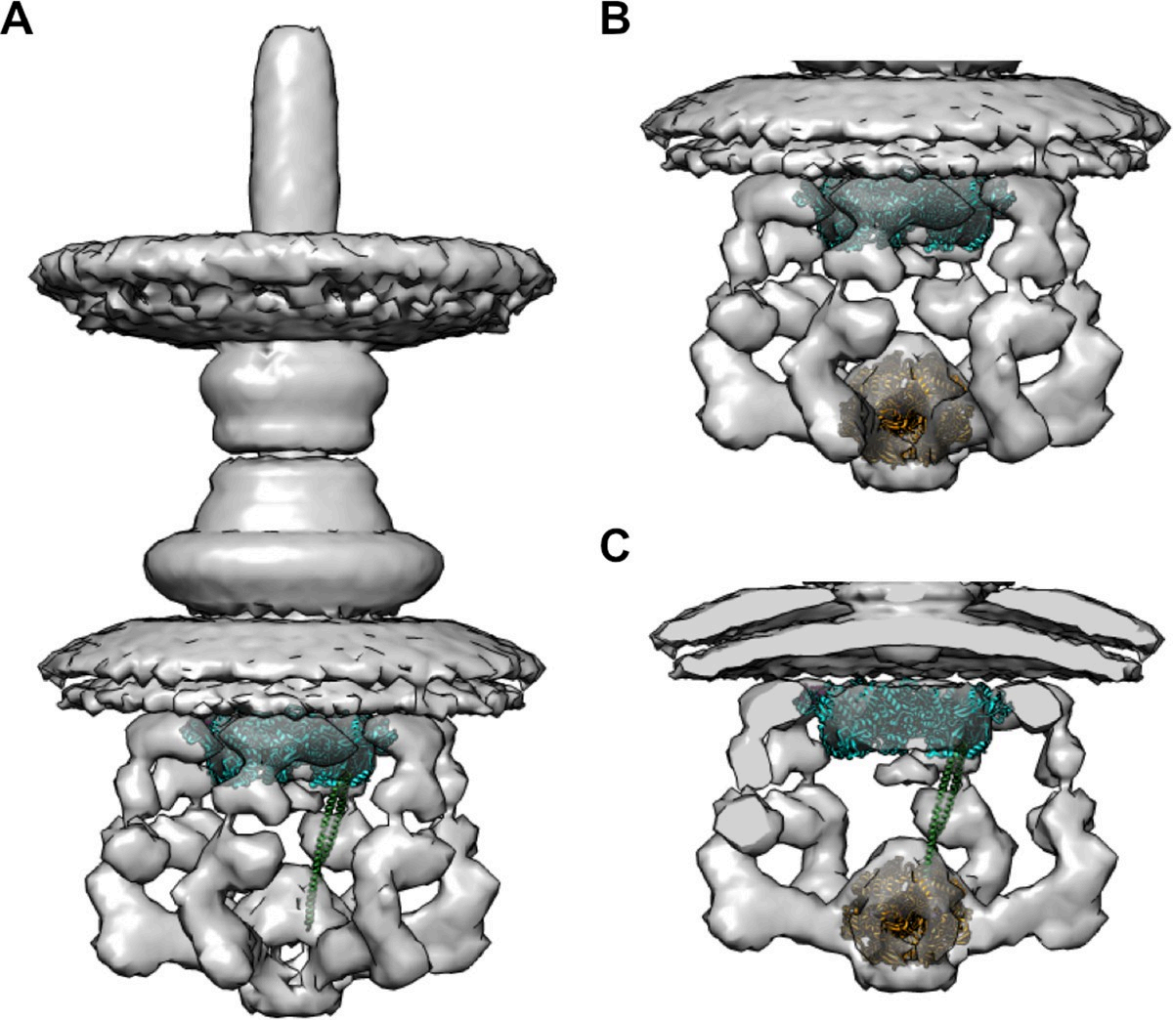
Overview of the export gate, central stalk, and ATPase proteins in the context of the entire T3SS cytoplasmic complex. CdsV_C_ (cyan) and full-length CdsO (green) were docked into the *Salmonella* injectisome map (EMDB 8544). (A) The extended CdsO structure easily spans the distance between export gate and the proposed location of the ATPase, penetrating the ATPase region by an additional ~ 50 Å. (B) Proposed sites of the export gate and ATPase (orange; EscN; PDB 6NJP). (C) Slab view of the cytoplasmic T3S complex map, fit with the CdsV_C_ ring, a single CdsO, and EscN.

The CdsV_C_:CdsO_25-110_ structure shows a symmetric 9:9 stoichiometry, as expected for a symmetric nonamer. It is also clear that only one CdsO can bind the ATPase at a given time [25]. The significance of the 9:9 stoichiometry may be that the ATPase bound CdsO can be directed toward any of the 9 available binding sites on CdsV. The modest affinity between CdsO and CdsV_c_ (28 μM) suggests that CdsV likely interacts *in vivo* with ATPase-bound CdsO. While no direct measurement for CdsO concentration in *Chlamydia* is known, it is not among the ~470 relatively abundant proteins assessed by quantitative mass spectrometry, and is likely less abundant than CdsV, which was observed [34]. We suggest that chaperone binding may increase the modest affinity between CdsV and CdsO such that the ATPase-bound CdsO preferentially engages CdsV already loaded with chaperone-cargo complexes. SctO proteins may promote cargo delivery by bridging the central “pore” of T3SS ATPases to the periphery of the export gate. The EscN:EscO cryo-EM structure shows a single EscO protruding from the asymmetric EscN hexamer at a ~70° angle [25], while CdsO_25-110_ exits the CdsV_C_ ring at an ~60° angle (S9E Fig). As shown in the EscN:EscO complex, lysines and arginines of the central stalk EscO directly interact with glutamate residues lining the pore of the ATPase EscN, which, concomitant with ATP hydrolysis, likely provide the rotational force of the ATPase to twist CdsO [25]. Given that, in the EscN:EscO structure, the two helices of the EscO coiled-coil are relatively equal in length, it is unknown how far a single helix of the central stalk may penetrate the ATPase in cases such as for CdsO, wherein the central stalk is asymmetric and, in general, longer than EscO. For F_1_- and V_1_-ATPase complexes, the central stalk extends around 70 Å into the catalytic core [20,21]. Manual modeling of an extended CdsO structure easily bridges the gap between the export gate platform and ATPase seen in the tomographic reconstruction from *Salmonella* (Fig 5 and [10]), with an additional ~50 Å situated within the density assigned to the ATPase (Fig 5C). Thus, both structures support SctO proteins connecting the ATPase pore with the periphery of the export gate.

Comparison of the structures of CdsV_C_ and the CdsV_C_: CdsO_25-110_ complex indicates that binding of CdsO to CdsV alters the adjacent binding site for chaperone-cargo complexes. We suggest, based on the rotational movement expected from the EscN-EscO structure [25], that the ATPase- catalyzed twisting of CdsO could release chaperone-cargo complexes. This would release substrates from the export gate by disrupting the SD2-4 cleft and might also create a pathway between subunits to the secretion pore. The FlhA:FliS and FlhA:FliT structures [18] show cargo binding to the periphery of the export gate such that the secretion is initiated by cargo entering the export gate from the periphery, which could be initiated by the ATPase twisting CdsO.

Reports of direct interactions of the export gate and ATPase complex have included the observation that the interface between FlhA and FliJ (CdsV and CdsO homologs) is mediated by conserved residues Phe72 and Leu76 of FliJ (S9C Fig). Mutations of these residues significantly reduced FliJ’s binding affinity for FlhA [31]. These residues instead likely serve to stabilize the FliJ coiled-coil. Manual docking of FliJ into the CdsO binding site of CdsV_C_ indicates that the closest sidechain, F72, is >6 Å from CdsV_C_ and pointing back toward the hydrophobic core of the FliJ coiled-coil (S9C Fig). Conversely, mutants within the same region of PscO, the CdsO homolog of *Pseudomonas aeruginosa*, upregulated secretion [35]. However, these residues lie lower along the PscO coiled-coil than the interaction interface that we have observed in our structure (S9D Fig). Additionally, mutation of several residues of FlhA have been shown to inhibit binding to FliJ, including FlhA residues E351, D356, R391, K392, K393, and L401 [19]. These residues align to subdomain 1 of CdsV_C_; thus, they are not directly involved in binding CdsO.

In summary, we present the atomic features and interaction interface of two critical components of the T3SS cytoplasmic sorting complex- the export gate CdsV with the ATPase’s central stalk protein CdsO. Suprisingly, CdsO does not engage the central pore of CdsV, but instead docks at a peripheral intersubunit interface and is positioned to create an opening between CdsV subunits allowing a route for bound cargo to enter the secretion apparatus. Further biophysical studies will be essential to describe how the energetics of ATP hydrolysis and the proton motive force are coupled to drive contraction and dilation of the export gate to promote virulence.

## Methods

### Expression and purification of CdsV_C_ and CdsO

CdsV residues 345–710 was amplified from *Chlamydia pneumoniae* and cloned into a pET28 expression vector, to utilize the vector’s N-terminal hexa-His-tag and thrombin cleavage site. Protein expression was performed at low temperature (18°C for 16 hours) in BL21 Star (DE3), after addition of 1 mM isopropyl β-d-thiogalactopyranoside for induction. Bacteria were collected by centrifugation and flash frozen in liquid nitrogen for later use. Bacteria were lysed with an Emulsiflex homogenizer (Avestin) in 25 mM sodium phosphate pH 8.0, 150 mM NaCl, with 10 μg/mL leupeptin, 1 μg/mL hen egg white lysozyme, 1 mM PMSF, 1 μg/mL DNase I and 0.7 μg/mL pepstatin. The lysate was clarified by centrifugation, and CdsV_C_ was purified with Talon metal affinity resin followed by gel-filtration in 10 mM HEPES pH 7.5, 150 mM NaCl (when proteins were prepared for ITC, 500mM NaCl was used). Nonameric peak fractions (CdsV_C_ is a mixture of monomer/dimer and nonomer during gel-filtration) were pooled and concentrated with an Amicon ultrafiltration cell to 2mg/ml for crystallization. The CdsV_C_ L638/D639A mutant was purified following the same protocol as for the WT CdsV_C_.

CdsO residues 25–110 were also amplified from *Chlamydia pneumoniae* and cloned into pET28. Protein expression and purification were performed as described above for CdsV, with the exception that the final gel-filtration buffer contained 300 mM NaCl.

### Crystallization and data collection

CdsV_C_ was crystallized by hanging drop vapor diffusion from a reservoir containing 100 mM HEPES pH 6.75 and 5% polyethylene glycol-6000 (PEG-6000), at 21°C. Crystals were obtained after ~ 2 weeks, cryoprotected using crystallization buffer supplemented with 20% glycerol, and cryo-cooled in liquid nitrogen. For heavy atom derivates, crystals were soaked in 1 mM heavy atoms in mother liquor for 2 days and harvested as for native crystals. X-ray data were collected at 100 K at LS-CAT Sector 21 at the Advance Photon Source (Argonne, IL). The data-collection statistics are given in Table 1. Diffraction intensities were processed and scaled with XDS [36]. Crystals were relatively non-isomorphous and an AuCl_2_-soaked crystal, with no evidence of bound gold, was used as a native. The data obtained from the crystals soaked in three heavy atoms- AuCl_3_, PtCl_4_ and UO_2_(CH_3_CO_2_)_2_, as well as an AuCl_3_ soaked “native” used as the input to SHARP [37] to solve the phase problem using Multiple Isomorphous Replacement (MIRAS). This led to the determination of 6 Pt-sites, 23 U-sites, and 27 Au-Sites by employing the MR-SAD program in Phenix [38,39]. Phasing and density modification using SHARP resulted in a clearly interpretable electron density map.

Crystallization CdsV-CdsO was performed using multiple CdsO constructs, with the final structure including CdsV_C_ and residues 25–110 of CdsO (CdsO_25-110_). CdsV_C_ and CdsO_25-110_ were mixed with ~10% molar excess of CdsO_25-110_ and crystals were grown from 100 mM Bis-Tris pH 6.0, 4% PEG 3350, and 200 mM ammonium acetate. The CdsV_C_ -CdsO_25-110_ structure was determined by molecular replacement using CdsV_C_ in Phenix.

### Structure determination and analysis

A partial model of CdsV was built in COOT [40] and used to identify non-crystallographic symmetry operators, which were then used in Phenix to perform further rounds of density modification. The complete structure was built using COOT, refined in Phenix, and evaluated against 2mFo-DFc and mFo-DFc maps calculated in Phenix. This structure was used as a search model with a non-isomorphous native dataset. Five percent of the reflections from all datasets were used for R_*free*_ sets.

Refinement was performed in Phenix, and included individual B-factors, TLS refinement, non-crystallographic symmetry restraints, and secondary structure restraints. Grouped B-factors were used in refinement of the CdsV_C_:CdsO complex. Refinement statistics are listed in Table 1. All figures were prepared using Pymol (v. 2.0, Schrodinger, LLC) or UCSF Chimera [41], ClustalW [42], the ConSurf server (https://consurf.tau.ac.il/), and the PISA server (http://www.ebi.ac.uk/pdbe/prot_int/pistart.html) [43]. Structure determination, analysis, and visualization software used were curated by SBGrid [44].

### Biophysical measurements

Isothermal titration calorimetry measurements were performed on a TA Instruments Nano ITC. Measurements were performed at 20°C with a 300 μL cell volume and 24 x 2 μL injections with a stirring rate of 150 rpm. All proteins were in 10 mM HEPES pH 7.5, 500 mM NaCl. The respective protein concentrations were 15.0 uM, 12.1 uM, and 2.2 mM for nonameric CdsVc, nonameric CdsVc L638A/D639A, and monomeric CdsO_25-110_. Calculations were performed using the TA instrument ITC Analyze software.

### Thermal unfolding

The stability of WT CdsV_C_ and the CdsV_C_ L638/D639A double mutant were assessed by thermal unfolding based on intrinsic tryptophan fluorescence. The WT and mutant CdsV_C_ proteins were diluted to 50 μM with gel filtration buffer and loaded into Tycho NT.6 capillaries (NanoTemper Technologies, Germany). Experiments were performed using a NanoTemper Tycho NT.6 instrument. The temperature gradient monitored was from 35 to 95°C, increasing by 0.5°C sec^-1^. Protein unfolding was recorded by measuring changes in tryptophan fluorescence at emission wavelengths of 330 and 350 nm as a function of temperature. Inflection temperatures were determined by automatic fitting of fluorescence ratios (350/330 nm) with a polynomial function, where the maximum slope corresponds to the peak of its first derivative.

### Generation of chromosomal mutations in *P*. *aeruginosa*

Primers specifying the alanine mutations at codons 635 and 636 (PcrD635A636A-3-1: 5'-GGCAGCTACCTGGCCGCCGCGCCGGCGATAACCCAGGCCTTC-3', PcrD635A636A-5-2: 5'-GAAGGCCTGGGTTATCGCCGGCGCGGCGGCCAGGTAGCTGCC-3') were paired with flanking primers (pcrDC5X 5’-AAAAAtctagagACCTTCCTGGCTCTCGCGCTGCT-3’, pcrDGFP-3-2 5’-AAAAAaagcttTCGTTCATGTCGCCCATGGTAGGGAT-3’) to generate flanks, which were subsequently joined by spicing by overlap extension PCR [45]. The construct to fuse two copies of the VSV-G epitope tag to the 5' end of the *pcrD* open reading frame was constructed by pairing primers pcrDVG2-3-1 (5-ATTAGGAAAAGTGTACACGGACATCGAGATGAACAGGTTGGGCAAAAACGACCTGAGCGGGCTTCTCG-3') and pcrDVG2-5-2 (5'-ATGTCCGTGTACACTTTTCCTAATCTATTCATTTCAATATCTGTATAGTTCATTCCCGCGCCTCCAGCTCCAGC-3') with flanking primers pcrDC5X (5'-AAAAAtctagagACCTTCCTGGCTCTCGCGCTGCT-3') and pcrD3H (5'-AAAAAaagcttTCACAACACGATCCTGCCAAGCGGCT-3', lower case indicates restriction sites used for cloning). Flanking PCR products were combined by splicing by overlap extension PCR. The PCR products specifying mutations were then cloned as XbaI/HindIII fragments into the allelic exchange vector pEXG2 [46]. The plasmids were moved into *P*. *aeruginosa* PAO1 *ΔexsE* [33] by mating, and the mutations were introduced into the chromosome by allelic exchange.

### Pseudomonas secretion assay

PAO1F Δ*exsE*, PAO1 Δ*exsE pcrD-VG2* and PAO1 Δ*exsE pcrD(L635A+D636A)-VG2* were grown in LB supplemented with 2.5 g/L NaCl to late log phase. Cultures were harvested and resuspended in 2 mL LB with or without 5 mM EGTA. After 30 min, 1 mL of culture was pelleted, and protein was precipitated from 500 uL of supernatant. The pellets were resuspended and normalized to a final OD_600_ of 2.5. Samples were separated by SDS-PAGE on a 10% gel (BioRad) and transferred to a PVDF membrane. With the exception of RpoA and VSV-G-tagged PcrD, the indicated proteins were detected by Western Blot using affinity purified rabbit antisera. RpoA was detected using a commercial mouse monoclonal antibody (BioLegend), and VSV-G using a commercial rabbit antibody (Thermo).

## Supporting information

S1 Table Residues modeled into electron density in the CdsV_C_ and CdsV_C_:CdsO structures. (DOCX)

S1 Fig Protein organization of CdsO and CdsV. For CdsO, residues corresponding to α-helix 1 and 2 are indicated, as are the transmembrane and cytoplasmic domains for CdsV. The residue numbers of the protein regions used in the experiments described in this paper are highlighted in red.
(TIF)

S2 Fig Evolutionarily conserved residues of the export gate apparatus. Sequence conservation displayed on the CdsV_C_ structure (on a scale from cyan (variable) to purple (conserved)), as determined by the ConSurf server, and based on the alignment of CdsV_C_ orthologs from *Pseudomonas*, *Shigella*, *Yersinia*, *Bordetella*, *Salmonella*, and *Vibrio* (S4 Fig). Surface representations of CdsV_C_ include (clockwise from upper left) bottom, top, lateral, and slab views.
(TIF)

S3 Fig Key residues that define the interface between two CdsV_C_ protomers. Residues corresponding to chain A are underlined. Chain A is colored as in Fig 1C.
(TIF)

S4 Fig Sequence conservation of CdsV_C_ orthologs. Primary sequence alignment of CdsV_C_ from *Chlamydia* (Uniprot ID Q9Z8L5), *Pseudomonas* (Q9I327), *Shigella* (P0A1I5), *Yersinia* (P0C2V3), *Bordetella* (Q84CT3), *Salmonella* (A0A0F7J9S2), and *Vibrio* (A0A2A2ND56). Residues involved in the CdsV_C_ oligomeric interface are highlighted in green. Conserved lysines and arginines that line the pore are indicated with red stars. Prolines 656 and 658 are highlighted in blue.
(TIF)

S5 Fig Electrostatic properties of the CdsV_C_ ring. Surface representations of the (clockwise from top left) bottom, top, lateral, and slab views of CdsV_C_, colored by electrostatic potential (red is negative, blue is positive), calculated using the APBS plug-in in PyMol. Conserved lysines and arginines that line the pore can be observed in the slab view (lower left).
(TIF)

S6 Fig Differences between the apo-CdsV_C_ and CdsO-bound CdsV_C_ structures. (A) Superposition of protomers from the apo-CdsV_C_ structure (middle) and the CdsV_C_:CdsO structure (right). Apo-CdsV_C_ is displayed to the left and colored by subdomain, for reference. (B) Helix 3 of subdomain 2 shifts toward subdomain 4 by ~5 Å when CdsV_C_ is bound to CdsO (cyan), as compared to the apo structure (magenta). (C) β-strands 8 and 11 adjust by ~2.3 and ~2.5 Å in the presence of CdsO.
(TIF)

S7 Fig Comparison of “open” CdsV with “open” and “closed” conformations of FlhA and MxiA. Despite the loops of subdomains 2 and 4 extending into the SD2-4 cleft (A-B), the unbound (A) and CdsO-bound CdsV (B) structures more closely align with the “open” conformation of FlhA, observed in both chaperone-bound forms (C-D). In the “closed” state of MxiA and FlhA (E-F), subdomains 2 and 4 dramatically shift to close the SD2-4 cleft. (A) Apo-CdsV, colored by subdomains as for Fig 1C; (B) CdsO-bound CdsV; (C) FliS-bound FlhA (6CH3 [18]); (D) FliT-bound FlhA (6CH2 [18]); (E) MxiA from *Shigella flexneri* (4A5P [26]); (F) FlhA from *Helicobacter pylori* (3MYD [30]).
(TIF)

S8 Fig Evolutionarily conserved and variable residues of CdsO orthologs. (A) Sequence alignment of CdsO from *Chlamydia* (Q9Z7J9) with orthologs from *Pseudomonas* (A0A0C6F691), *Shigella* (P0A1K3), *Yersinia* (A0A0E1NFR4), *Bordetella* (A0A0E8FIJ9), *Salmonella* (P0A1K2), *Vibrio* (A0A0H6WY40), and *Escherichia* (B7UMA5). Thr 67, the residue at the center of the loop connecting the two helices of the CdsO coiled-coil, is indicated with a black star. (B) Representative cartoon of CdsO determined in this work, colored according to sequence conservation (using ConSurf), with an extended model of CdsO, shown as both cartoon and surface representation. More conserved residues are located near the N- and C-termini of the coiled-coil. (C) An extended model of CdsO, colored by electrostatic potential (red is negative, blue is positive). The two views are obtained by 180° rotation about the y-axis.
(TIF)

S9 Fig Model of the export gate:central stalk:ATPase interaction. (A) Using the *Chlamydia trachomatis* CdsO structure (3K29) as a guide, residues of CdsO not visible in our crystal structure were modeled in COOT (colored in grey). (B) Comparison of the CdsV_C_-bound CdsO (left) with CdsO from *C*. *trachomatis* (blue; PDB 3K29; [31]); FliJ from *Salmonella* (orange; 3AJW; [1]); and YscO from *Vibrio* (purple; 4MH6). (C) The structure of FliJ manually docked into the CdsO binding site of CdsV, with FliJ residues proposed to influence export gate binding and secretion [48] shown as sticks. (D) The structure of YscO manually docked into the CdsO binding site of CdsV, with residues that impact secretion shown as sticks [35]. The corresponding residues from PscO are in parentheses. (E) Angles between the extension of CdsO from CdsV_C_ and EscO from EscN from the crystal structure and cryo-EM structures (6NJP), respectively, are noted.
(TIF)

S10 Fig Biophysical characterization of the L638A/D639A mutant on thermal stability and affinity toward CdsO_25-110_. (A) and (B) show representative isothermal titration calorimetry traces for CdsVc and the L638A/D639A mutant titrated with CdsO_25-110_. (A) CdsVc binds CdsO_25-110_ with a Kd of 28 ± 3 μM and displays an exothermic isotherm. The L638A/D639A mutant does not show detectible binding toward CdsO_25-110_ an has an endothermic isotherm. (C) Thermal unfolding curves for CdsVc and the L638A/D639A mutant, revealing that both are quite stable with melting temperatures of 58°C and 55 C°, respectively.
(TIF)

S11 Fig CdsV_C_ and CdsV_C_:CdsO crystal packing. Top and side views, with the unit cell, of the crystal packing of CdsV_C_ (A) and CdsV_C_:CdsO (B).
(TIF)
