# Supplementary material for: “The structure of the Type III secretion system export gate with CdsO, an ATPase lever arm”
Source: PLoS Pathog. 2020 Oct 13;16(10):e1008923. doi: 10.1371/journal.ppat.1008923 (PMC7584215; doi:10.1371/journal.ppat.1008923)
Supplement: S1 Table — (DOCX) [file ppat.1008923.s001.docx]

**Supporting Information.**

**S1 Table. Residues mapped to electron density in the CdsV_C_ and CdsV_C_:CdsO structures**.

| Chain ID | CdsV_C_ | CdsV_C_:CdsO |
| --- | --- | --- |
| A | 363 – 710 | 364 – 610 |
|  |  | 614 – 625 |
|  |  | 634 – 700 |
| B | 363 – 709 | 362 – 709 |
| C | 361 – 453 | 362 – 708 |
|  | 458 - 657 |  |
|  | 661 - 709 |  |
| D | 363 – 658 | 363 – 462 |
|  | 662 – 708 | 468 – 610 |
|  |  | 615 – 700 |
|  |  | 707 – 708 |
| E | 363 – 658 | 362 – 708 |
|  | 662 – 708 |  |
| F | 363 – 624 | 364 – 709 |
|  | 630 – 707 |  |
| G | 364 – 627 | 362 – 710 |
|  | 631 – 709 |  |
| H | 362 – 623 | 362 – 705 |
|  | 640 – 656 |  |
|  | 663 – 703 |  |
| I | 362 – 629 | 362 – 469 |
|  | 640 - 658 | 477 - 708 |
|  | 662 - 708 |  |
| L |  | 23 – 105 |
| M |  | 36 – 100 |
| N |  | 25 – 110 |
| O |  | 33 – 106 |
| P |  | 36 – 100 |
| Q |  | 34 – 104 |
| R |  | 42 – 100 |
| S |  | 45 – 84 |
| T |  | 28 - 102 |
