# Supplementary figures and images for: “The structure of the Type III secretion system export gate with CdsO, an ATPase lever arm”
Source: PLoS Pathog. 2020 Oct 13;16(10):e1008923. doi: 10.1371/journal.ppat.1008923 (PMC7584215; doi:10.1371/journal.ppat.1008923)

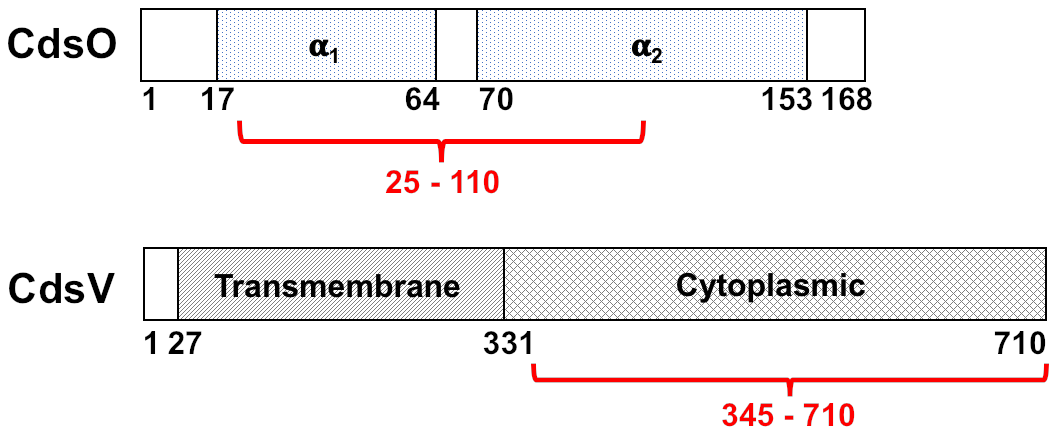

Supplement: S1 Fig — For CdsO, residues corresponding to α-helix 1 and 2 are indicated, as are the transmembrane and cytoplasmic domains for CdsV. The residue numbers of the protein regions used in the experiments described in this paper are highlighted in red. (TIF) [file ppat.1008923.s002.tif]

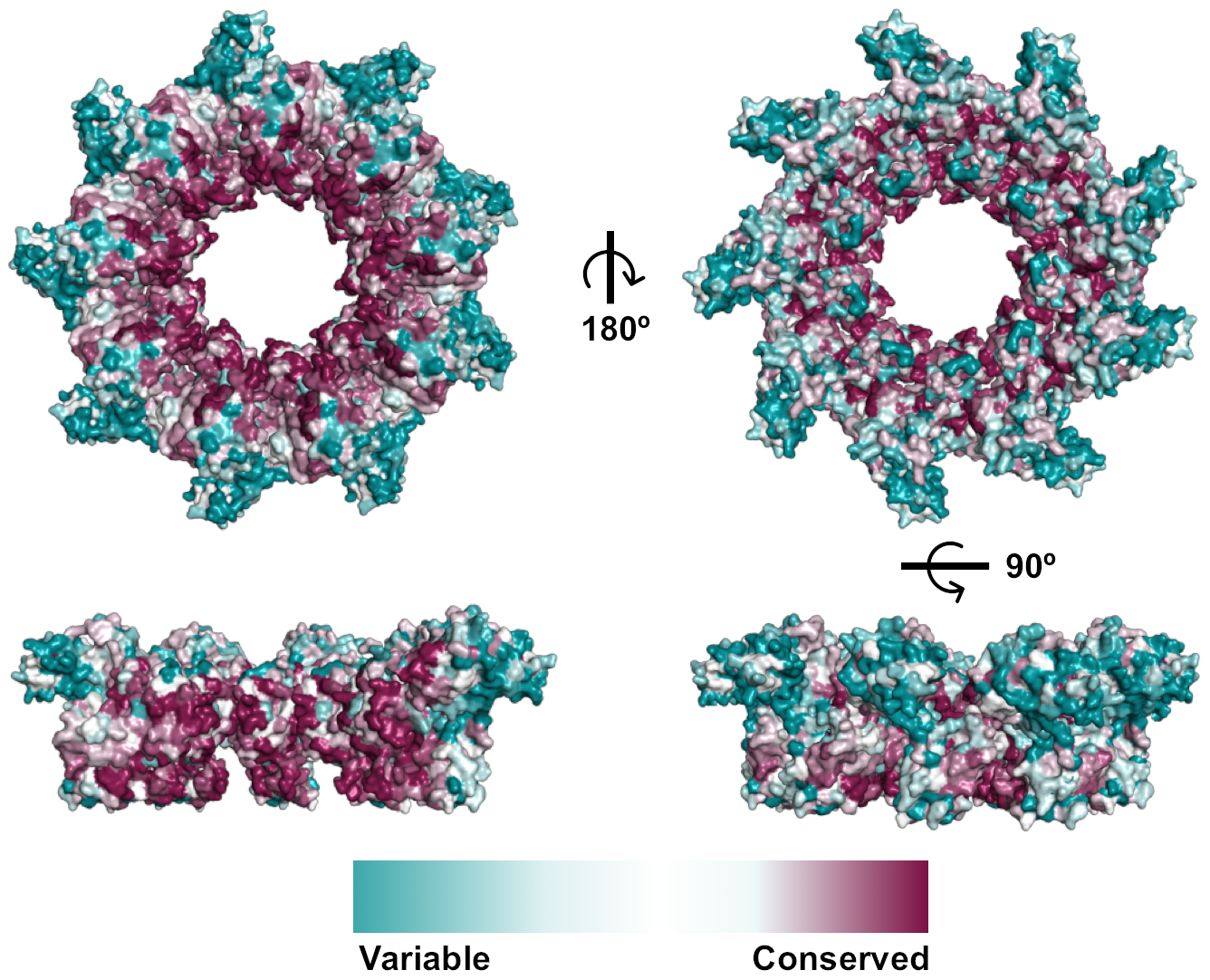

Supplement: S2 Fig — Sequence conservation displayed on the CdsVC structure (on a scale from cyan (variable) to purple (conserved)), as determined by the ConSurf server, and based on the alignment of CdsVC orthologs from Pseudomonas, Shigella, Yersinia, Bordetella, Salmonella, and Vibrio (S4 Fig). Surface representations of CdsVC include (clockwise from upper left) bottom, top, lateral, and slab views. (TIF) [file ppat.1008923.s003.tif]

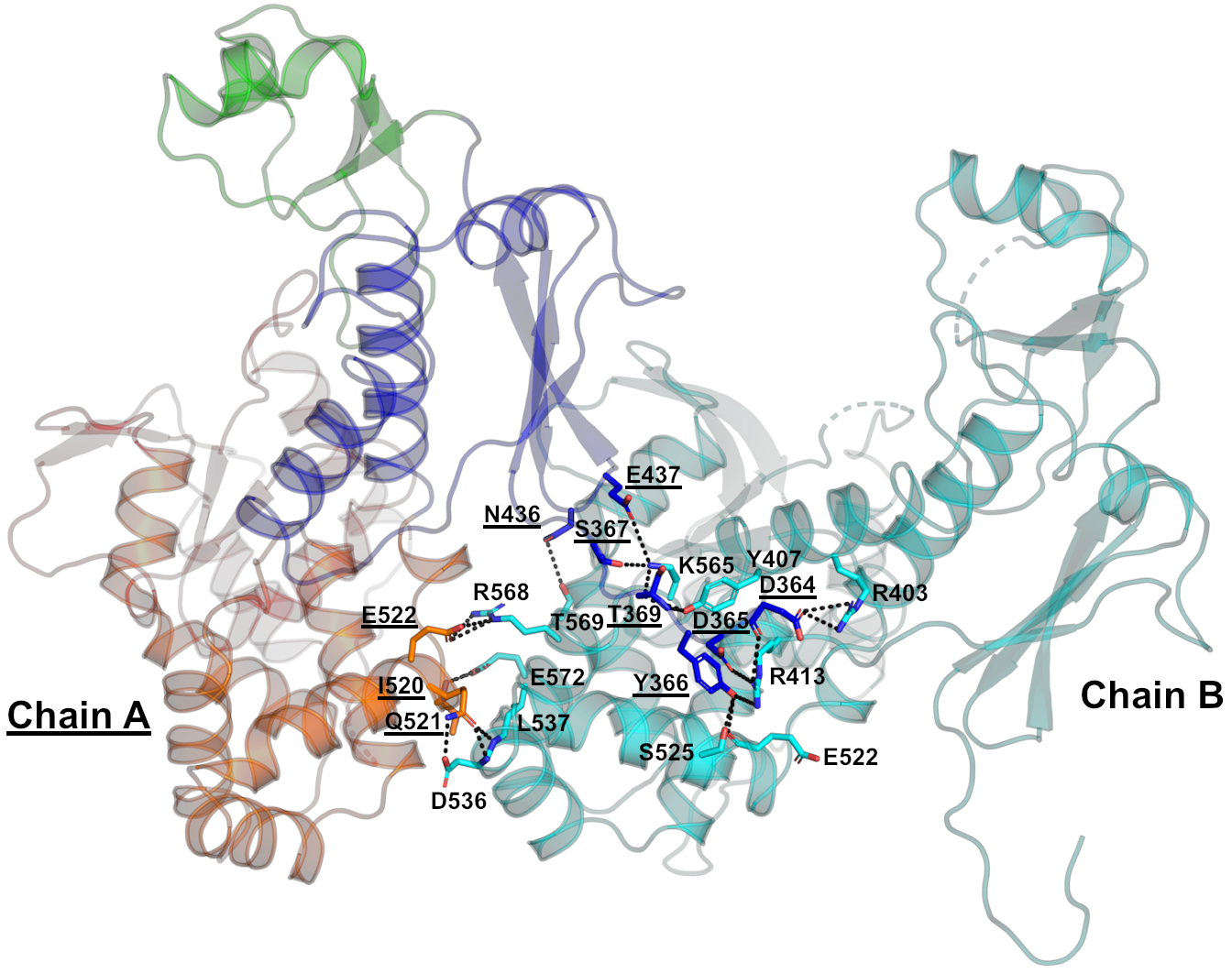

Supplement: S3 Fig — Residues corresponding to chain A are underlined. Chain A is colored as in Fig 1C. (TIF) [file ppat.1008923.s004.tif]

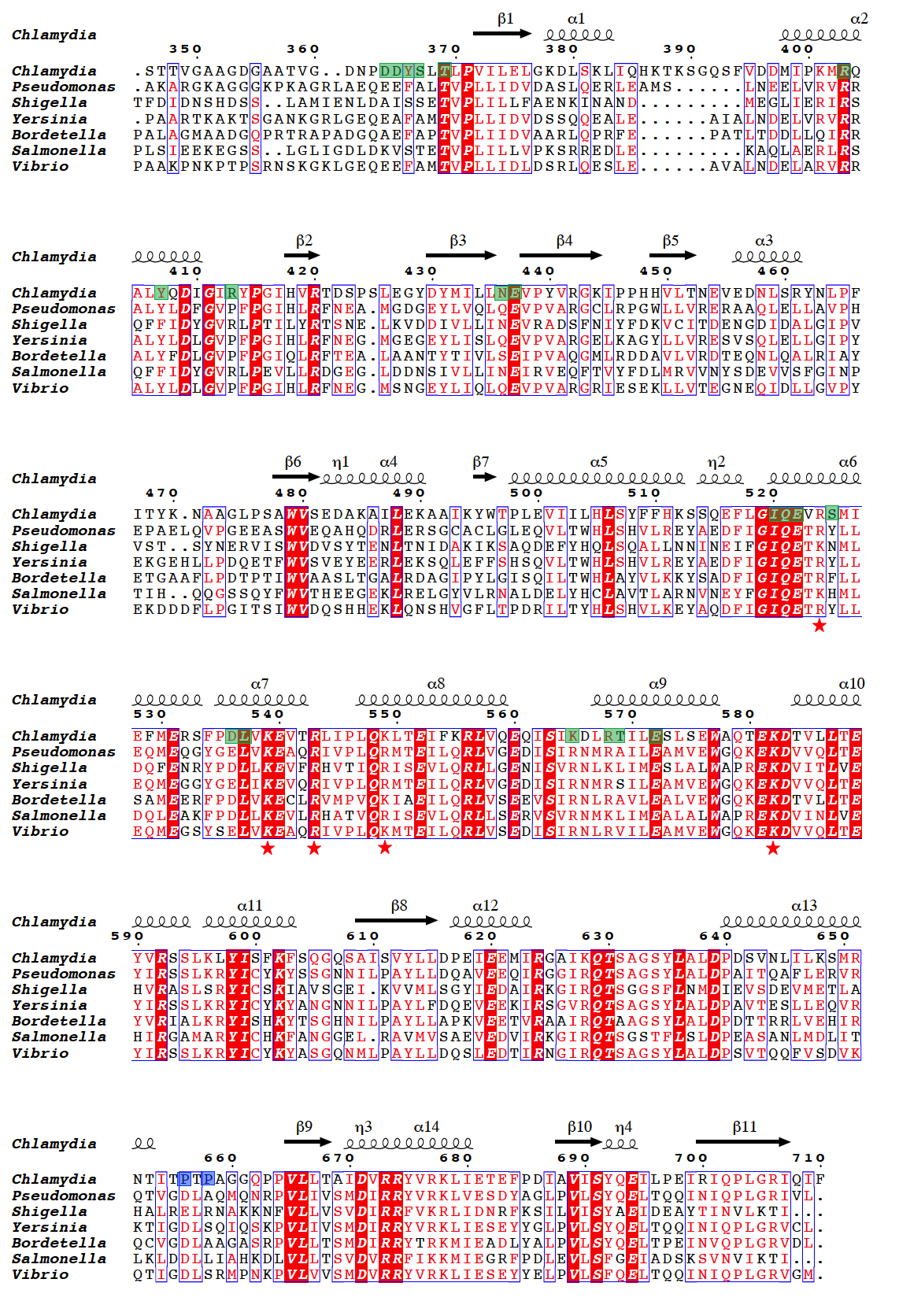

Supplement: S4 Fig — Primary sequence alignment of CdsVC from Chlamydia (Uniprot ID Q9Z8L5), Pseudomonas (Q9I327), Shigella (P0A1I5), Yersinia (P0C2V3), Bordetella (Q84CT3), Salmonella (A0A0F7J9S2), and Vibrio (A0A2A2ND56). Residues involved in the CdsVC oligomeric interface are highlighted in green. Conserved lysines and arginines that line the pore are indicated with red stars. Prolines 656 and 658 are highlighted in blue. (TIF) [file ppat.1008923.s005.tif]

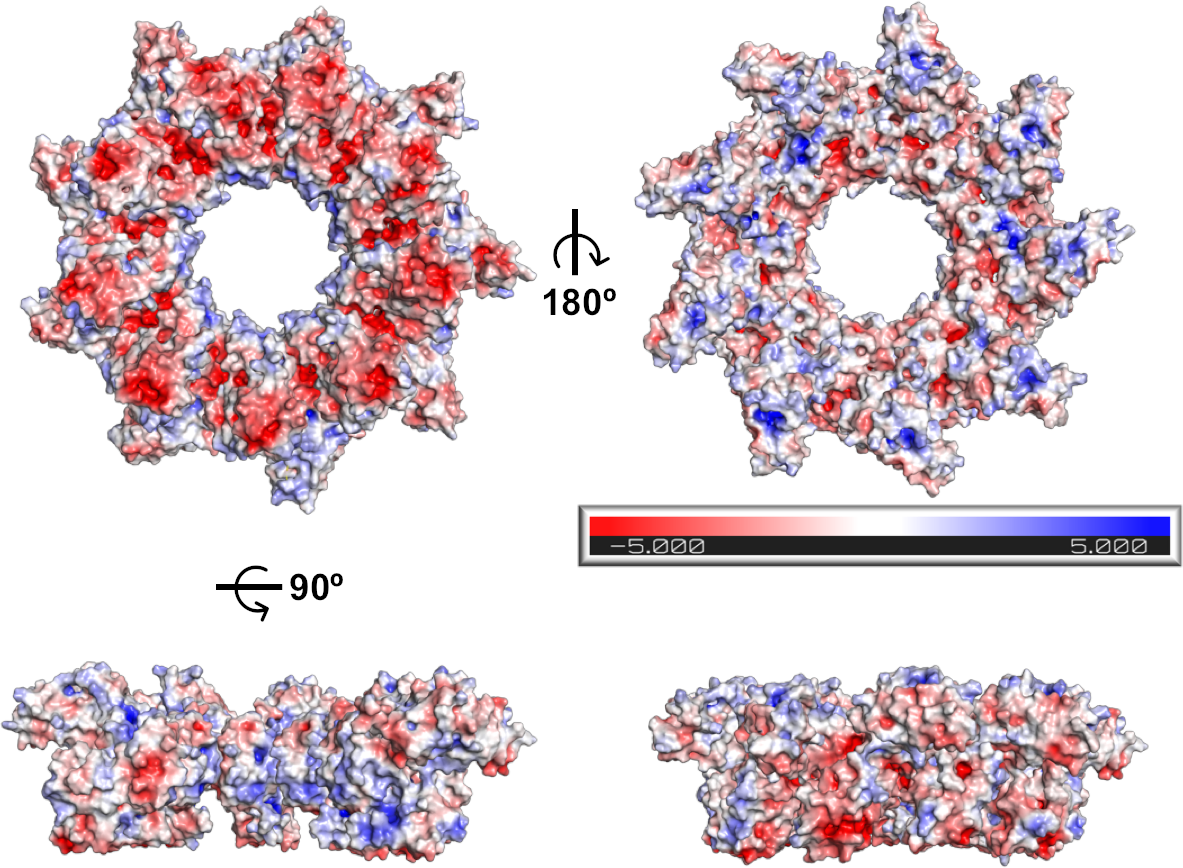

Supplement: S5 Fig — Conserved lysines and arginines that line the pore can be observed in the slab view (lower left). (TIF) [file ppat.1008923.s006.tif]

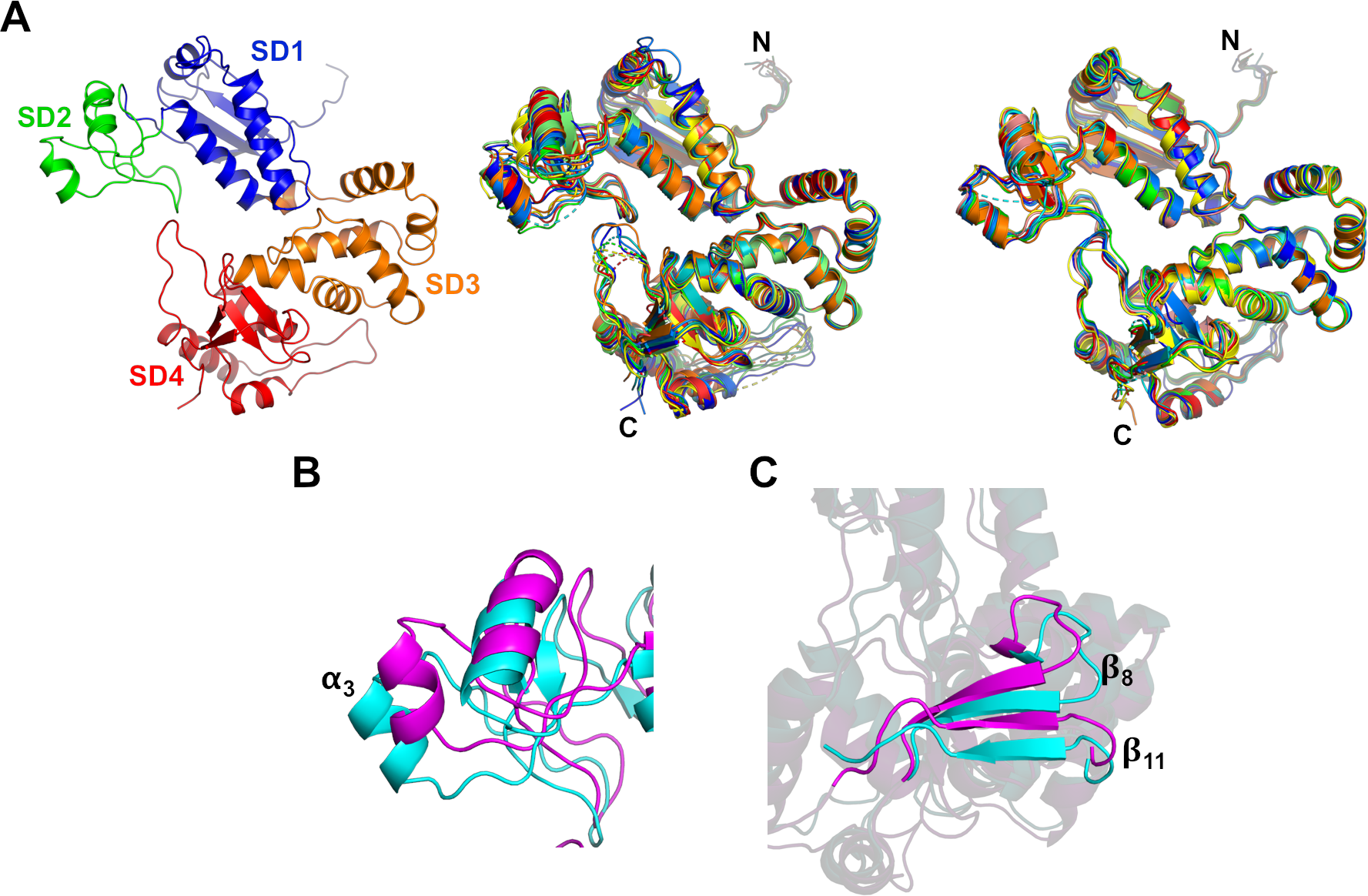

Supplement: S6 Fig — (A) Superposition of protomers from the apo-CdsVC structure (middle) and the CdsVC:CdsO structure (right). Apo-CdsVC is displayed to the left and colored by subdomain, for reference. (B) Helix 3 of subdomain 2 shifts toward subdomain 4 by ~5 Å when CdsVC is bound to CdsO (cyan), as compared to the apo structure (magenta). (C) β-strands 8 and 11 adjust by ~2.3 and ~2.5 Å in the presence of CdsO. (TIF) [file ppat.1008923.s007.tif]

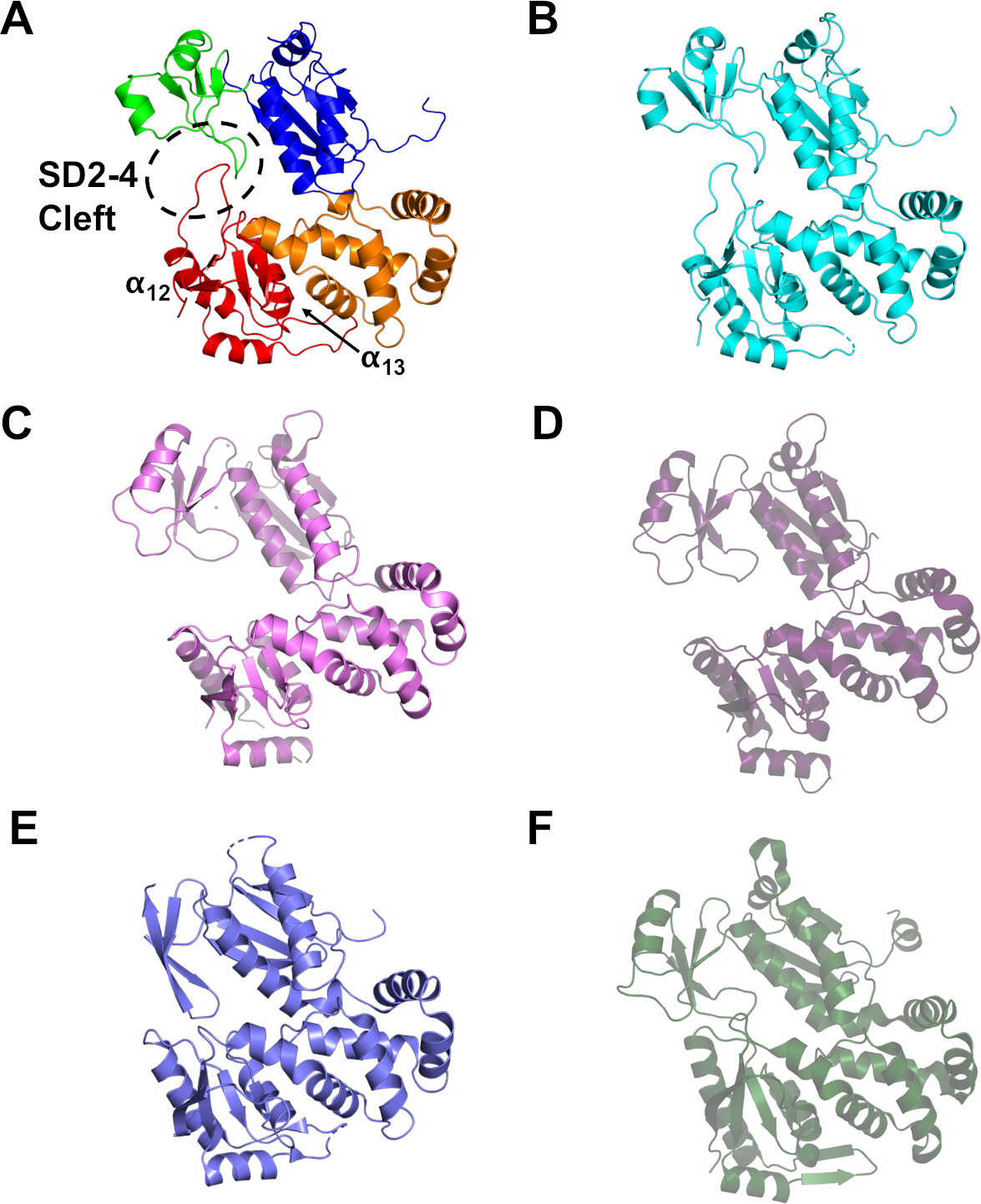

Supplement: S7 Fig — Despite the loops of subdomains 2 and 4 extending into the SD2-4 cleft (A-B), the unbound (A) and CdsO-bound CdsV (B) structures more closely align with the “open” conformation of FlhA, observed in both chaperone-bound forms (C-D). In the “closed” state of MxiA and FlhA (E-F), subdomains 2 and 4 dramatically shift to close the SD2-4 cleft. (A) Apo-CdsV, colored by subdomains as for Fig 1C; (B) CdsO-bound CdsV; (C) FliS-bound FlhA (6CH3 [18]); (D) FliT-bound FlhA (6CH2 [18]); (E) MxiA from Shigella flexneri (4A5P [26]); (F) FlhA from Helicobacter pylori (3MYD [30]). (TIF) [file ppat.1008923.s008.tif]

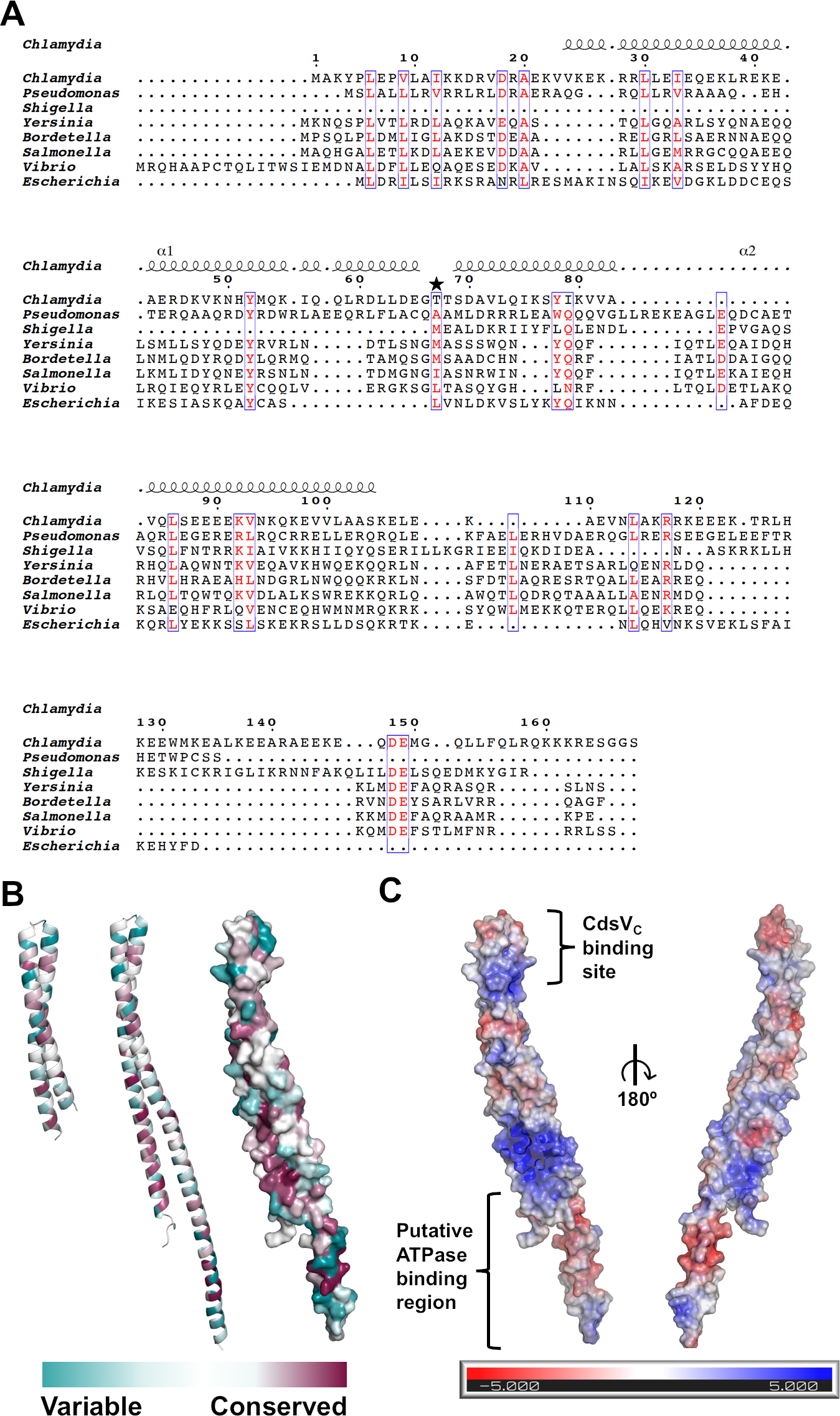

Supplement: S8 Fig — (A) Sequence alignment of CdsO from Chlamydia (Q9Z7J9) with orthologs from Pseudomonas (A0A0C6F691), Shigella (P0A1K3), Yersinia (A0A0E1NFR4), Bordetella (A0A0E8FIJ9), Salmonella (P0A1K2), Vibrio (A0A0H6WY40), and Escherichia (B7UMA5). Thr 67, the residue at the center of the loop connecting the two helices of the CdsO coiled-coil, is indicated with a black star. (B) Representative cartoon of CdsO determined in this work, colored according to sequence conservation (using ConSurf), with an extended model of CdsO, shown as both cartoon and surface representation. More conserved residues are located near the N- and C-termini of the coiled-coil. (C) An extended model of CdsO, colored by electrostatic potential (red is negative, blue is positive). The two views are obtained by 180° rotation about the y-axis. (TIF) [file ppat.1008923.s009.tif]

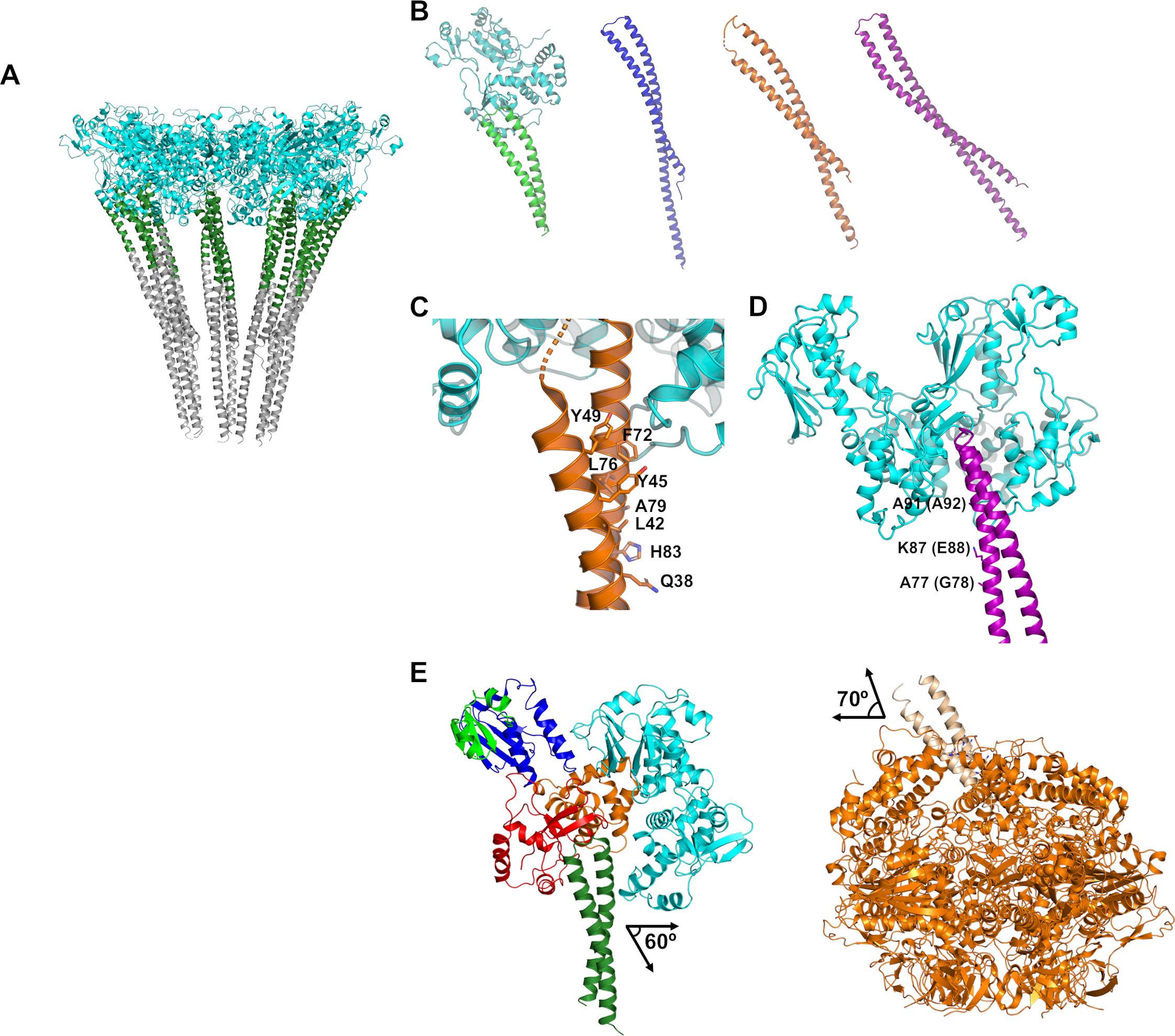

Supplement: S9 Fig — (A) Using the Chlamydia trachomatis CdsO structure (3K29) as a guide, residues of CdsO not visible in our crystal structure were modeled in COOT (colored in grey). (B) Comparison of the CdsVC-bound CdsO (left) with CdsO from C. trachomatis (blue; PDB 3K29; [31]); FliJ from Salmonella (orange; 3AJW; [1]); and YscO from Vibrio (purple; 4MH6). (C) The structure of FliJ manually docked into the CdsO binding site of CdsV, with FliJ residues proposed to influence export gate binding and secretion [48] shown as sticks. (D) The structure of YscO manually docked into the CdsO binding site of CdsV, with residues that impact secretion shown as sticks [35]. The corresponding residues from PscO are in parentheses. (E) Angles between the extension of CdsO from CdsVC and EscO from EscN from the crystal structure and cryo-EM structures (6NJP), respectively, are noted. (TIF) [file ppat.1008923.s010.tif]

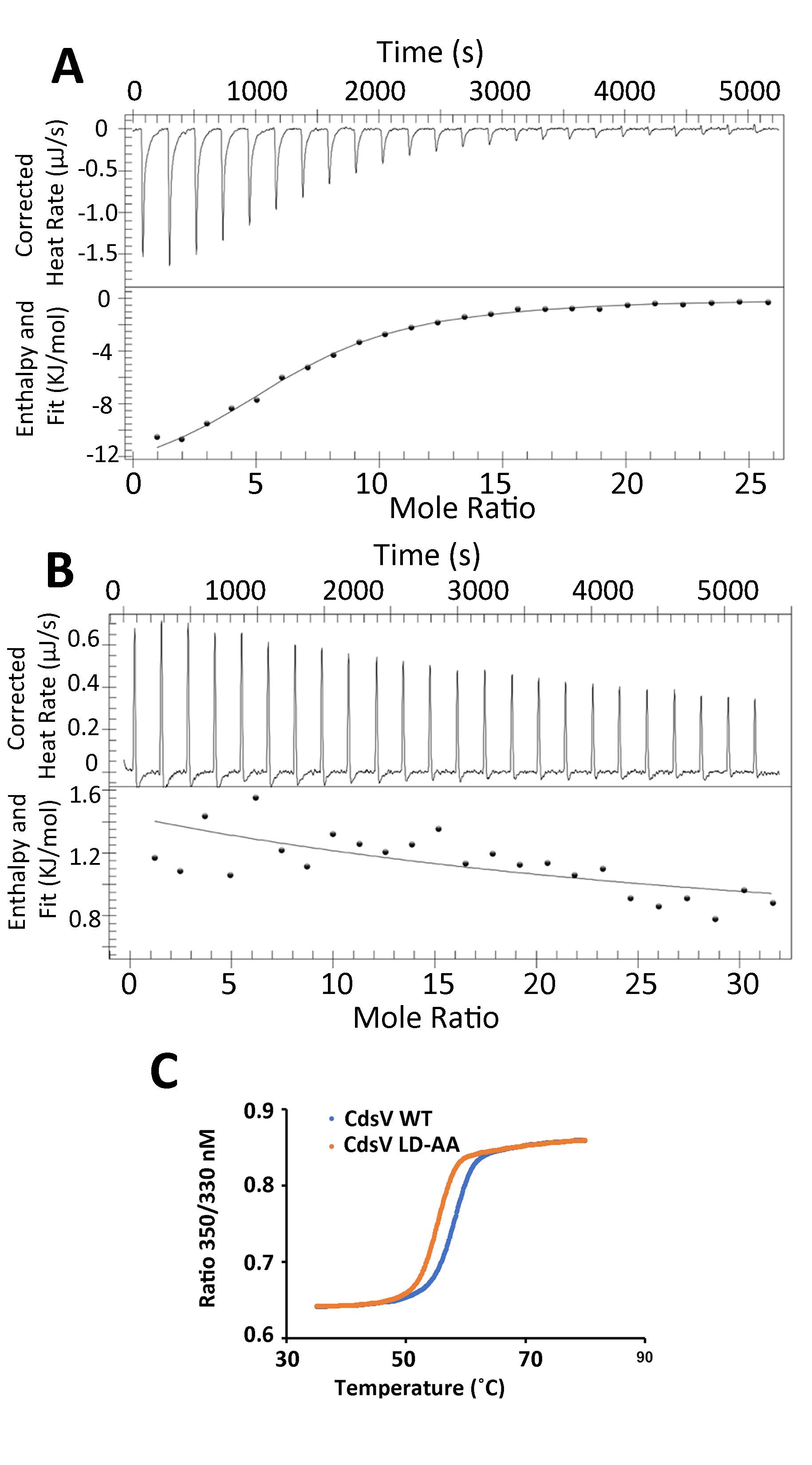

Supplement: S10 Fig — (A) and (B) show representative isothermal titration calorimetry traces for CdsVc and the L638A/D639A mutant titrated with CdsO25-110. (A) CdsVc binds CdsO25-110 with a Kd of 28 ± 3 μM and displays an exothermic isotherm. The L638A/D639A mutant does not show detectible binding toward CdsO25-110 an has an endothermic isotherm. (C) Thermal unfolding curves for CdsVc and the L638A/D639A mutant, revealing that both are quite stable with melting temperatures of 58°C and 55 C°, respectively. (TIF) [file ppat.1008923.s011.tif]

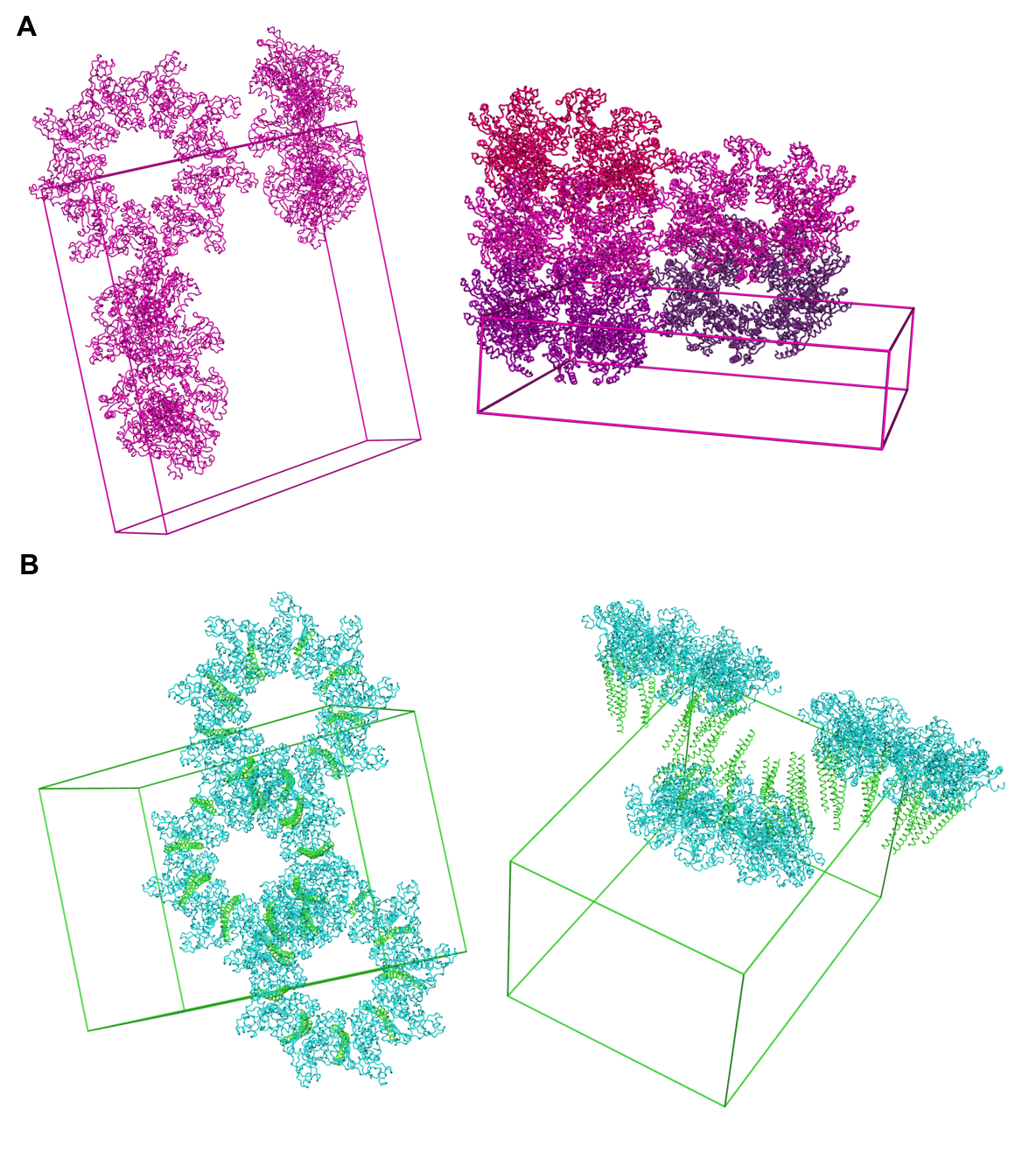

Supplement: S11 Fig — Top and side views, with the unit cell, of the crystal packing of CdsVC (A) and CdsVC:CdsO (B). (TIF) [file ppat.1008923.s012.tif]
